# Membrane lipid and expression responses of *Saccharolobus islandicus* REY15A to acid and cold stress

**DOI:** 10.3389/fmicb.2023.1219779

**Published:** 2023-08-15

**Authors:** Beverly K. Chiu, Jacob Waldbauer, Felix J. Elling, Öykü Z. Mete, Lichun Zhang, Ann Pearson, Erin M. Eggleston, William D. Leavitt

**Affiliations:** ^1^Department of Earth Sciences, Dartmouth College, Hanover, NH, United States; ^2^Department of the Geophysical Sciences, The University of Chicago, Chicago, IL, United States; ^3^Department of Earth and Planetary Sciences, Harvard University, Cambridge, MA, United States; ^4^Leibniz-Laboratory for Radiometric Dating and Isotope Research, Kiel University, Kiel, Germany; ^5^Department of Biology, Middlebury College, Middlebury, VT, United States; ^6^Department of Chemistry, Dartmouth College, Hanover, NH, United States

**Keywords:** archaea, GDGT, cold stress, acid stress, *Saccharolobus islandicus*, thermoacidophile, stress response

## Abstract

Archaea adjust the number of cyclopentane rings in their glycerol dibiphytanyl glycerol tetraether (GDGT) membrane lipids as a homeostatic response to environmental stressors such as temperature, pH, and energy availability shifts. However, archaeal expression patterns that correspond with changes in GDGT composition are less understood. Here we characterize the acid and cold stress responses of the thermoacidophilic crenarchaeon *Saccharolobus islandicus* REY15A using growth rates, core GDGT lipid profiles, transcriptomics and proteomics. We show that both stressors result in impaired growth, lower average GDGT cyclization, and differences in gene and protein expression. Transcription data revealed differential expression of the GDGT ring synthase *grsB* in response to both acid stress and cold stress. Although the GDGT ring synthase encoded by *grsB* forms highly cyclized GDGTs with ≥5 ring moieties, *S. islandicus grsB* upregulation under acidic pH conditions did not correspond with increased abundances of highly cyclized GDGTs. Our observations highlight the inability to predict GDGT changes from transcription data alone. Broader analysis of transcriptomic data revealed that *S. islandicus* differentially expresses many of the same transcripts in response to both acid and cold stress. These included upregulation of several biosynthetic pathways and downregulation of oxidative phosphorylation and motility. Transcript responses specific to either of the two stressors tested here included upregulation of genes related to proton pumping and molecular turnover in acid stress conditions and upregulation of transposases in cold stress conditions. Overall, our study provides a comprehensive understanding of the GDGT modifications and differential expression characteristic of the acid stress and cold stress responses in *S. islandicus*.

## 1. Introduction

Many archaea produce membrane-spanning isoprenoid lipids called glycerol dibiphytanyl glycerol tetraethers (GDGTs) that are unique to the Archaeal domain of life (Pearson and Ingalls, 2013; Schouten et al., 2013; Villanueva et al., 2014). Individual GDGTs may contain 0–8 cyclopentyl ring moieties denoted as GDGT-0 through GDGT-8 (De Rosa et al., 1980a; De Rosa and Gambacorta, 1988). It has been proposed that archaea modify the rigidity and permeability of their membranes by modifying the numbers of cyclopentyl rings in their GDGTs, where GDGTs with a higher number of cyclopentyl rings can pack together more tightly to decrease membrane permeability and increase heat stability (Gliozzi et al., 1983; Gabriel and Chong, 2000; Shinoda et al., 2005). By adjusting membrane stability and permeability, archaea demonstrate homeoviscous adaptation in response to environmental stressors (Oger and Cario, 2013). Previous experiments showed that shifts in temperature, pH, or energy availability each caused thermoacidophilic archaea to alter the average cyclization of their membrane GDGTs (e.g., De Rosa et al., 1980a; Zillig et al., 1983; De Rosa and Gambacorta, 1988; Boyd et al., 2011; Jensen S. et al., 2015; Feyhl-Buska et al., 2016; Cobban et al., 2020; Quehenberger et al., 2020; Zhou et al., 2020). Despite these observations, the underlying expression systems leading to changes in GDGT cyclization as part of the archaeal stress response are poorly understood.

Few studies have examined how archaeal transcript and protein expression correlate with changes in membrane GDGT composition, due in part to the fact that enzymes for several key steps in the GDGT biosynthesis pathway were only recently discovered. These enzymes include a calditol synthase (Cds) that synthesizes calditol headgroups important for acidic pH conditions, a tetraether synthase (Tes) required for tetraether formation, and GDGT ring synthases (GrsAB) responsible for cyclopentyl ring formation (Zeng et al., 2018, 2019, 2022; Lloyd et al., 2022). GrsA and GrsB are homologous radical SAM enzymes that successively insert cyclopentyl rings at distinct positions on the GDGT core lipid (Zeng et al., 2019). GrsA introduces cyclopentyl rings at C-7 positions forming GDGTs 1 through 4, followed by GrsB activity at C-3 positions forming GDGTs 5 through 8 (Zeng et al., 2019). The protein abundance of GrsA and the transcript and protein abundance of GrsB showed pH and temperature dependence supporting a temporal expression model of the GDGT ring synthases that enables *Sulfolobus acidocaldarius* to modify its GDGT cyclization under environmental fluctuations (Yang et al., 2023). With the characterization of GrsA and GrsB, we can now examine how the expression of GDGT ring synthases corresponds with changes in GDGT cyclization of other archaea facing environmental stressors.

Here we performed parallel acid and cold stress experiments with *Saccharolobus islandicus* REY15A, hypothesizing that these stresses would induce changes in core GDGT cyclization, transcriptomes and proteomes. *S. islandicus* (formerly known as *Sulfolobus islandicus*) is a genetically tractable model thermoacidophilic archaeon in the order *Sulfolobales* (Zhang et al., 2018; Counts et al., 2021). We chose *S. islandicus* REY15A as our study organism because its genome has been described, and we expected its lipid profile to be responsive to environmental stresses and growth phase based on a prior study with another strain of *S. islandicus* (Guo et al., 2011; Jensen S. et al., 2015). While *S. islandicus* produces both diether and tetraether lipids with various polar headgroups that can contribute to membrane adaptation, we focus our efforts on core GDGTs specifically (Oger and Cario, 2013; Jensen S. M. et al., 2015). No prior study to our knowledge has combined transcriptomics or proteomics with GDGT profiling to characterize the stress response of *S. islandicus* to changes in temperature or pH. The *S. islandicus* REY15A genome contains single copies of both *grsA* and *grsB*, which simplifies expression analysis of these genes and their encoded proteins in relation to changes in core GDGT cyclization. Overall, we characterize the physiological, proteomic, transcriptomic, and core lipid responses of *S. islandicus* to acid and cold stress, with targeted analysis of lipid biosynthesis genes. These observations inform a broader understanding of *Sulfolobales* cellular stress response to major environmental parameters in their hot and acidic natural habitats.

## 2. Materials and methods

### 2.1. Growth experiments

**FIGURE 1:**
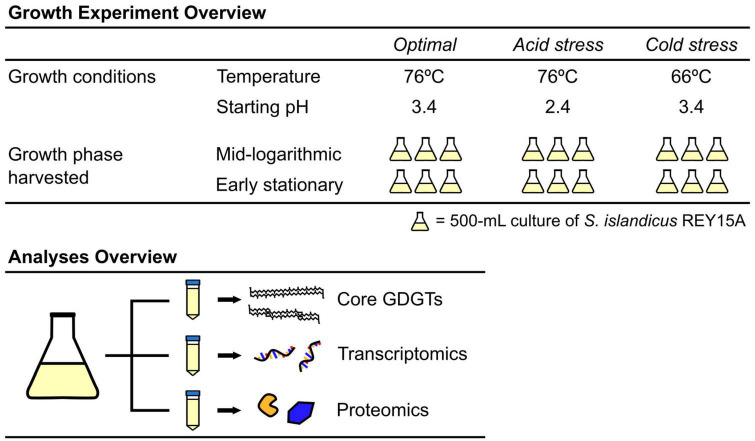
Overview of the growth experiments and downstream analyses, illustrating the conditions corresponding to the 18 samples of *S. islandicus* REY15A from which all lipid and expression data in this study were derived.

We performed experiments with *S. islandicus* strain REY15A under three conditions defined by different pH and temperature combinations (Figure 1). These included the optimal growth condition control (pH 3.4, 76°C), an acid stress condition (pH 2.4, 76°C), and a cold stress condition (pH 3.4, 66°C). All cultures were grown as batch cultures in 1-L Erlenmeyer flasks containing 500-ml DY medium (Zhang et al., 2013) with D-arabinose (Sigma-Aldrich, USA) as the carbon and energy source in place of dextrin, hereafter AY medium. Cultures were incubated in Innova-42 shaking incubators (Eppendorf, Hauppauge, NY, USA) at 150 RPM and 66 or 76°C. To minimize culture evaporation at these high incubation temperatures, open trays of water were maintained in the incubator to ensure a saturated atmosphere. Culture flasks had loose-fitting plastic caps to allow for continuous atmospheric oxygen exchange with the media. We monitored culture growth by optical density measurements at 600 nm (OD_600_) on a Genesys 10S UV-Vis spectrophotometer (Thermo Scientific, Waltham, MA, USA). OD_600_ data were used to calculate growth rates using a rate calculation script from Cobban et al. (2020) and available on GitLab.^1^ Calculations of growth rates for each condition only incorporated OD_600_ data from the triplicate cultures designated for early stationary phase harvest given that monitoring of mid-log-designated cultures ceased after harvest.

**FIGURE 2:**
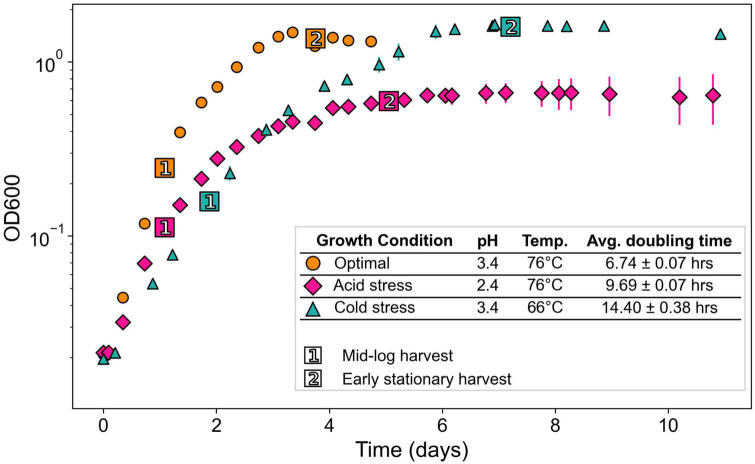
Growth curves of *S. islandicus* from all tested growth conditions. All series are the average of triplicates and error bars represent standard error. (Inset) Growth conditions with resulting growth rates and lipid ring index values. Temp., temperature; ML, mid-logarithmic phase; ES, early stationary phase.

Experiments for each growth condition consisted of one uninoculated control and six inoculated replicates. Cultures were inoculated with actively growing pre-cultures to starting optical densities (OD_600_) of 0.02. Triplicate cultures were harvested at timepoints corresponding to mid-log and early stationary phase of each growth condition (Figure 2). Harvest timepoints were based on preliminary growth experiments characterizing *S. islandicus* growth under each growth condition (data not shown). For each harvest, biomass samples from the triplicate cultures were sacrificed, processed, and preserved for core lipid analysis, RNA sequencing, and proteomics (Figure 1). Given the anticipated biomass requirements for each downstream analysis and the variable OD_600_ of each growth phase and condition, sampling volumes differed between experiments and are summarized in Supplementary Table 1. We also measured culture pH at each harvest to constrain average pH drifts over the course of growth under all conditions (Supplementary Table 1).

For each sampling time point, we prioritized biomass for RNA-seq as follows. The harvested culture was immediately mixed with an equal volume of chilled, sterile basal salts medium (AY medium without arabinose or tryptone) and placed on ice until centrifugation. Biomass pellets were collected by centrifuging the culture:basal salts solution for 18 min in an Eppendorf centrifuge with swinging bucket rotor set at 3,163 × *g* (max speed) and 4°C, and decanting the resulting supernatant. Pellets were immediately placed on dry ice and then transferred to a −80°C freezer until RNA extraction. In parallel, to preserve biomass for proteomics and lipid extractions, harvested culture was centrifuged (3,163 × *g*, 4°C, 18 min) and decanted, and resulting pellets were frozen in a −80°C freezer until further processing.

### 2.2. Lipid analysis

Frozen biomass pellets of *S. islandicus* harvested from growth experiments and designated for lipid extraction were freeze-dried prior to lipid extraction. We obtained core GDGT fractions from freeze-dried biomass using acid hydrolysis and solvent extraction as described previously, with modified 3N methanolic HCl incubation conditions of 65°C for 90 min (Zhou et al., 2020). Given that calditol-linked GDGTs found in *Sulfolobales* are resistant to hydrolysis by the methanolic HCl method used here, our results only include non-calditol core GDGTs. GDGT abundances were measured by ultra-high-performance liquid chromatography (UHPLC) coupled to an Agilent 6410 triple-quadrupole mass spectrometer (MS) as originally described by Becker et al. (2013) and previously implemented for related organisms (Zhou et al., 2020). To enable analysis of small sample aliquots, data was collected using a Selected Ion Method (SIM) for core GDGTs only. The resulting peak areas of GDGTs in each sample were used to calculate ring indices (RI) according to Eq. 1. Like previous studies of *S. acidocaldarius* (Cobban et al., 2020; Zhou et al., 2020), multiple isomers of GDGTs 3–5 were detected in some *S. islandicus* samples. Peak areas of minor isomers were summed with their respective major components before RI calculation.


$$\mathrm{RI}=\frac{\begin{array}{c} 1^{*}\left[\mathrm{GDGT}-1\right]+2^{*}\left[\mathrm{GDGT}-2\right]+3^{*}\left[\mathrm{GDGT}-3\right]\;\\ +4^{*}\left[\mathrm{GDGT}-4\right]+5^{*}\left[\mathrm{GDGT}-5\right]+6^{*}\left[\mathrm{GDGT}-6\right]\;\\ +7^{*}\left[\mathrm{GDGT}-7\right]+8^{*}\left[\mathrm{GDGT}-8\right]\; \end{array}}{\begin{array}{c} \left[\mathrm{GDGT}-1\right]+\left[\mathrm{GDGT}-2\right]+\left[\mathrm{GDGT}-3\right]\;\\ +\left[\mathrm{GDGT}-4\right]+\left[\mathrm{GDGT}-5\right]+\left[\mathrm{GDGT}-6\right]\;\\ +\left[\mathrm{GDGT}-7\right]+\left[\mathrm{GDGT}-8\right]\; \end{array}}\,\left(1\right)$$

Two sample *t*-tests were used to identify significant differences (*p*-value < 0.05) in RI and individual GDGT abundances between growth conditions.

### 2.3. RNA-seq

#### 2.3.1. RNA isolation

*Saccharolobus islandicus* RNA was extracted using RNeasy Midi or Mini kits (Qiagen, Hilden, Germany) with modifications based on published methods (Buetti-Dinh et al., 2016). Briefly, frozen biomass pellets were defrosted on wet-ice, resuspended in 1 ml lysis buffer, transferred to tubes containing 0.5 mm glass beads, then subjected to three cycles of freeze-thaw with bead beating to aid in cell lysis. We followed kit instructions for the remainder of the RNA extraction. For samples extracted with RNeasy Midi kits, we used our maximum centrifugation speed (3,163 × *g*) for all steps and increased centrifugation time of cell lysate through the filter column to 25 min and the Buffer RW1 wash step to 8 min.

Total RNA was treated with a TURBO DNA-free DNase treatment kit (Invitrogen, Waltham, MA, USA). RNA was confirmed pure by Nanodrop (Thermo Scientific, Waltham, MA, USA) measurements, quantified by Qubit RNA HS assay (Invitrogen), and checked for quality on a Lonza FlashGel RNA cassette gel (Lonza, Basel, Switzerland). Extracted RNA samples were stored at −80°C and mailed on dry-ice to the Joint Genome Institute for library preparation and sequencing.

#### 2.3.2. Library preparation and sequencing

The Joint Genome Institute (CA, USA) performed RNA library preparation and sequencing. Briefly, rRNA was removed from 10 ng of total RNA using Qiagen FastSelect 5S/16S/23S for bacterial rRNA depletion (and additional FastSelect plant and/or yeast rRNA depletion) (Qiagen) with RNA blocking oligo technology. The fragmented and rRNA-depleted RNA was reverse transcribed to create first strand cDNA using Illumina TruSeq Stranded mRNA Library prep kit (Illumina) followed by the second strand cDNA synthesis which incorporated dUTP to quench the second strand during amplification. The double stranded cDNA fragments were polyA-tailed and ligated to JGI dual indexed Y-adapters, followed by an enrichment of the library by 10 cycles of PCR. The prepared libraries were quantified using KAPA Biosystems’ next-generation sequencing library qPCR kit and run on a Roche LightCycler 480 real-time PCR instrument. Sequencing of the flowcell was performed on the Illumina NovaSeq sequencer using NovaSeq XP V1 reagent kits, S4 flowcell, following a 2x151 indexed run protocol.

#### 2.3.3. RNA-seq data processing and differential gene expression analysis

RNA-seq data were processed by JGI according to their RNA-Seq gene expression analysis pipeline as described here. The JGI QC pipeline was used for initial filtering and trimming of raw fastq file reads. Using BBDuk,^2^ raw reads were evaluated for artifact sequence by kmer matching (kmer = 25), allowing 1 mismatch and detected artifact was trimmed from the 3′ end of the reads. RNA spike-in reads, PhiX reads and reads containing any Ns were removed. Quality trimming was performed using the phred trimming method set at Q6. Following trimming, reads under the length threshold (25 bases or $\frac{1}{3}$ of the original read length, whichever was longer) were removed.

Filtered reads from each library were aligned to the *S. islandicus* REY15A reference genome (GenBank accession no. CP002425) using HISAT2 version 2.2.0 (Guo et al., 2011; Kim et al., 2015). Strand-specific coverage bigWig files (fwd and rev) were generated using deepTools v3.1 (Ramírez et al., 2014). featureCounts (Liao et al., 2014) was used to generate the raw gene counts file using gff3 annotations. Only primary hits assigned to the reverse strand were included in the raw gene counts (-s 2 -p –primary parameters). Raw gene counts were used to evaluate the level of correlation between biological triplicates from each growth condition at each growth phase using Pearson’s correlation. All replicates had Pearson correlation coefficients >0.9 within replicate group. One replicate from the cold stress condition at early stationary phase had relatively poor correlation within its replicate group with 0.71 as the highest correlation coefficient. However, this replicate was still used for downstream differential gene expression analysis as it did not have a correlation coefficient >0.71 + 0.05 with any libraries outside of its replicate group.

DESeq2 (version 1.30.0) (Love et al., 2014) was used to perform differential gene expression analysis between pairs of growth conditions. There were triplicate biological replicate datasets associated with every combination of growth condition and growth phase. To analyze the response of *S. islandicus* to acidic pH stress and cold temperature stress, we only analyzed the differential expression results from pairwise comparisons of each stress growth condition to optimal growth condition at the same growth phase (e.g., acidic pH stress at mid-log growth phase vs. optimal growth condition at mid-log growth phase). We considered genes to be differentially transcribed between pairs of conditions if they showed at least twofold change (log_2_ fold change ≤−1 or ≥1) and had adjusted *p*-values < 0.05 (default *p*-value adjustment by DESeq2 analysis using the Benjamini–Hochberg method). Negative log_2_ fold change (log2FC) values for transcripts indicate increased expression in the given stress condition relative to optimal conditions while positive log2FC values indicate decreased expression in the given stress condition.

### 2.4. Proteomics

#### 2.4.1. Protein extraction and LC/MS analysis

Cell pellets were extracted by vortexing and heating (95°C, 20 min) in a reducing and denaturing SDS (1%) / Tris (200 mM, pH 8.0) / DTT (10 mM) buffer, and cysteine thiols alkylated with 40 mM iodoacetamide. Proteins were purified by a modified eFASP (enhanced filter-aided sample preparation) protocol (Erde et al., 2014), using Sartorius Vivacon 500 concentrators (30 kDa nominal cut-off). Proteins were digested with MS-grade trypsin (37°C, overnight), and peptides were eluted from the concentrator dried by vacuum centrifugation. For quantitative analysis, peptides were isotopically labeled at both N- and C-termini using the diDO-IPTL methodology (Waldbauer et al., 2017). Briefly, C-termini were labeled with either oxygen-16 or -18 by enzymatic exchange in isotopic water of >98 atom % enrichment. N-termini were labeled with either un- or dideuterated formaldehyde via reductive alkylation using sodium cyanoborohydride. Peptide extracts from each sample were split and aliquots labeled separately with CD_2_O/^16^O and CH_2_O/^18^O; the latter were pooled to serve as a common internal standard for quantification. Aliquots of the ^16^O-labeled peptides and ^18^O-labeled internal standard were mixed 1:1 v/v and analyzed by LC-MS for protein expression quantification.

For LC-MS analysis, peptide samples were separated on a monolithic capillary C18 column (GL Sciences Monocap Ultra, 100 μm I.D. × 200 cm length) using a water-acetonitrile + 0.1% formic acid gradient (2–50% AcN over 180 min) at 360 nl/min using a Dionex Ultimate 3000 LC system with nanoelectrospray ionization (Proxeon Nanospray Flex source). Mass spectra were collected on an Orbitrap Elite mass spectrometer (Thermo) operating in a data-dependent acquisition (DDA) mode, with one high-resolution (120,000 *m*/Δ*m*) MS1 parent ion full scan triggering Rapid-mode 15 MS2 CID fragment ion scans of selected precursors. Proteomic mass spectral data were analyzed using MorpheusFromAnotherPlace (MFAP; Waldbauer et al., 2017), using the predicted proteome of *S. islandicus* REY15A as a search database. Precursor and product ion mass tolerances for MFAP searches were set to 20 ppm and 0.6 Da, respectively. Static cysteine carbamidomethylation and variable methionine oxidation, N-terminal (d4)-dimethylation, and C-terminal ^18^O_2_ were included as modifications. False discovery rate for peptide-spectrum matches was controlled by target-decoy searching to <0.5%. Protein-level relative abundances and standard errors were calculated in *R* using the Arm post processing scripts for diDO-IPTL data (Waldbauer et al., 2017).^3^

#### 2.4.2. Differential protein abundance analysis

Significantly differential protein expression between stressed and optimal conditions at each growth phase was determined by calculating a *Z*-score for protein abundance differences by taking the difference in the mean (log_2_-transformed) protein abundance between conditions and dividing it by the sum of the total uncertainty estimate for that protein in the two conditions. This total uncertainty estimate for a given condition was taken as the root-square sum of the standard deviation of a protein’s abundance across the biological replicates of that condition plus the average standard error of the protein’s abundance across quantified spectra within each replicate. *Z*-scores were converted to *p*-values assuming a standard normal distribution and then the familywise error rate for significantly differential expression between conditions was controlled by adjusting *p*-values to correct for multiple testing (Benjamini and Hochberg, 1995) with a threshold of adjusted *p* < 0.1. For consistency with our differential transcript expression analysis, we also imposed a minimum threshold of at least a twofold change (| log2FC| ≥ 1). Negative log2FC values for proteins indicate decreased abundance in the given stress condition relative to optimal conditions while positive log2FC values indicate increased abundance in the given stress condition. Proteomic mass spectral data are available via proteomeXchange under accession PXD037641 and the MassIVE repository^4^ under accession MSV000090562 (username for reviewers: MSV000090562_reviewer / password: rey15a).

## 3. Results

### 3.1. Acid and cold stress conditions impair *S. islandicus* growth

Acid and cold stress impaired *S. islandicus* growth relative to optimal conditions (Figure 2). Optimal growth conditions yielded the fastest average doubling time observed: 6.8 ± 0.1 h. Doubling times under acidic pH and cold temperature stress conditions were 9.8 ± 0.1 and 14.0 ± 0.5 h, respectively. Maximum culture densities also differed among growth conditions (Figure 2). Cultures grown under optimal conditions reached an average maximum optical density of 1.48 ± 0.06 while acidic pH and cold temperature cultures reached average maximum optical densities of 0.75 ± 0.11 and 1.69 ± 0.04, respectively. An ANOVA of average maximum cell densities indicated that at least one mean significantly differed from the others (*p* < 0.001), and a *post-hoc* Tukey HSD test showed that the acidic pH cultures had a significantly lower maximum cell density than optimal or cold temperature cultures.

### 3.2. *Saccharolobus islandicus* GDGT distributions change in response to acid and cold stress

**FIGURE 3:**
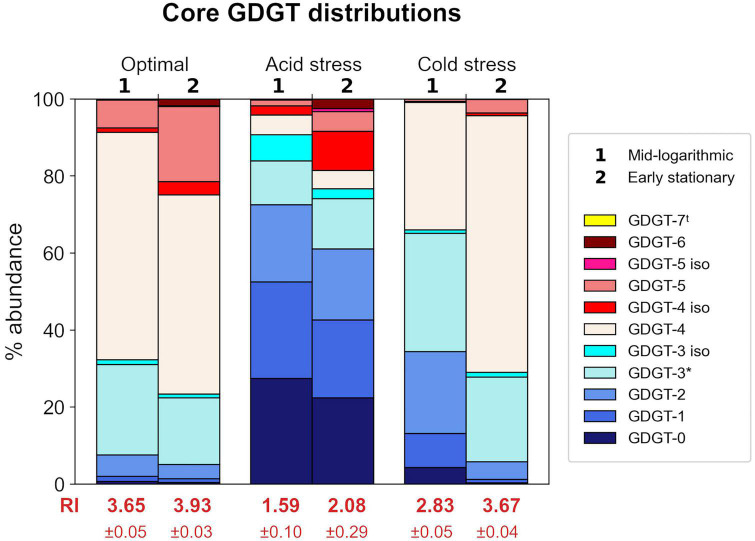
Relative abundances of core GDGTs from each growth condition at each time point harvested. Each stacked bar chart represents the average of triplicates. Ring Index (RI) values corresponding to each profile are indicated in red text. Isomers are indicated by “iso” in legend. ^t^GDGT-7 was detected in some samples, but only in small amounts <1% relative abundance. *GDGT-3 abundances are based on peak areas inclusive of a shoulder observed in corresponding chromatogram peaks.

*Saccharolobus islandicus* produced GDGT-0 through -6 in all experimental conditions, while average GDGT cyclization differed among growth conditions and growth phases (Figure 3). Small amounts of GDGT-7 occurred in profiles from optimal and acid stress conditions, but its relative abundance never exceeded 1% (Supplementary Table 2). As previously observed in *S. acidocaldarius*, we also detected the late eluting isomers (denoted by ′ symbol) of GDGTs 3–5 in *S. islandicus* lipid profiles (Cobban et al., 2020; Zhou et al., 2020). Isomers were particularly abundant (up to 10% relative abundance) in GDGT profiles from acid stress conditions (Supplementary Table 2). The average cyclization of GDGTs, as interpreted using Ring Index (RI) values, ranged from 1.59 to 3.93 for each combination of growth condition and growth phase (Figure 3). Average RI values significantly increased from mid-log phase to early stationary phase in optimal and cold stress conditions, indicating greater GDGT cyclization during later growth (Figure 3). Average RI increased at later growth under acid stress as well, but not significantly (*p* = 0.09).

Both acid stress and cold stress induced significantly decreased average RI values relative to optimal conditions at each growth phase (Figure 3). At mid-log growth phase, *S. islandicus* grown under optimal conditions had an average RI value of 3.65 ± 0.05 while acid and cold stress resulted in average RI values of 1.59 ± 0.10 and 2.83 ± 0.05, respectively. Similarly at early stationary phase, optimal conditions yielded an average RI value of 3.93 ± 0.03 while acid and cold stress resulted in average RI values of 2.08 ± 0.29 and 3.67 ± 0.04, respectively. Though both stressors resulted in lower RI, comparison of GDGT profiles resulting from each stress show that acid and cold conditions induced distinct changes of different individual GDGT abundances (significance determined by *t*-test). For example, cold stress GDGT profiles showed decreased relative abundances of all detected GDGTs with ≥5 rings while GDGT-5 was the only higher-numbered ring showing decreased abundance in acid stress profiles (Figure 3). Unlike cold stress, the decrease in RI under acid stress conditions was driven largely by increased abundances of GDGTs 0–2 and a dramatic reduction of GDGT-4 (Figure 3). Differences in the underlying GDGT abundance changes induced by each stress condition highlights the value of interpreting complete lipid profiles in addition to RI values.

### 3.3. RNA-seq

We obtained a total of 108.4 million raw reads from the 18 *S. islandicus* RNA samples derived from our growth experiments. Raw reads per sample averaged at 6.0 million (range: 3.7–10.1 million). After quality filtering and mapping fragments to the *S. islandicus* REY15A genome, an average of 91% (range: 65–95%) of fragments could be assigned to genes. Complete read and mapping data can be found in Supplementary Table 3.

**FIGURE 4:**
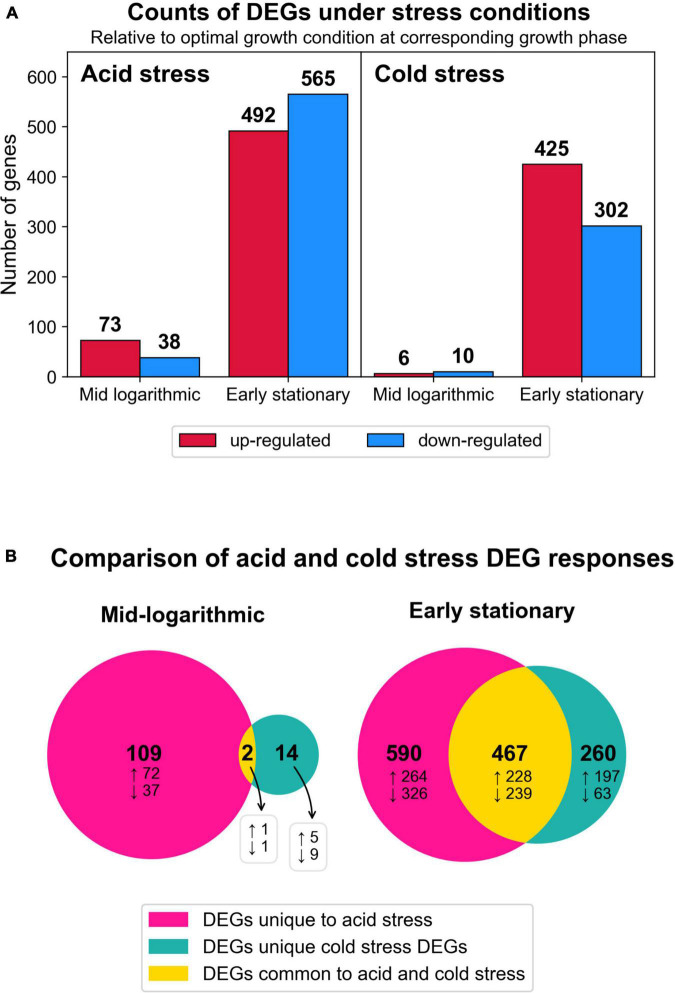
Summary of transcriptomic changes. **(A)** Counts of differentially expressed genes (DEGs) (log_2_ fold change ≥1, adjusted *p* < 0.05) in response to both stress conditions at each time point harvested. All results are relative to optimal growth conditions at corresponding growth phase. **(B)** Venn diagrams depicting the numbers of DEG responses (transcript identity and direction of change) that were unique to a given stress condition or common to both stresses tested. Total numbers of DEG responses are in bold text and the subset of DEG responses that were up or downregulated are denoted by up and down arrows, respectively.

To understand how *S. islandicus* transcriptomes responded to acid and cold stress, we compared the RNA-seq data from either stress condition versus optimal growth conditions as our control for baseline expression. Differential gene expression analysis showed that acid stress and cold stress each induced differentially expressed genes (DEGs) at both sampled growth phases though more DEGs occurred at early stationary phase in both stress conditions (Figure 4A).

We compared the DEGs from both stresses by growth phase and directionality (up or downregulated) to examine the number of DEG responses that were unique to either stress or common to both stresses in this study (Figure 4B). At both growth phases and for both expression directions, acid stress always yielded the highest proportion of observed DEGs (Figure 4B). At early stationary phase, a significant proportion of DEGs were commonly induced by both acid and cold stress, potentially representing more generalized stress responses by *S. islandicus* to environmental perturbations. Complete differential gene expression results are available in Supplementary Table 4. We present specific RNA-seq results with an initial focus on GDGT biosynthesis-related DEGs followed by an overview of broader DEG responses grouped by inducing stressor(s).

#### 3.3.1. Differentially expressed GDGT biosynthesis genes

**FIGURE 5:**
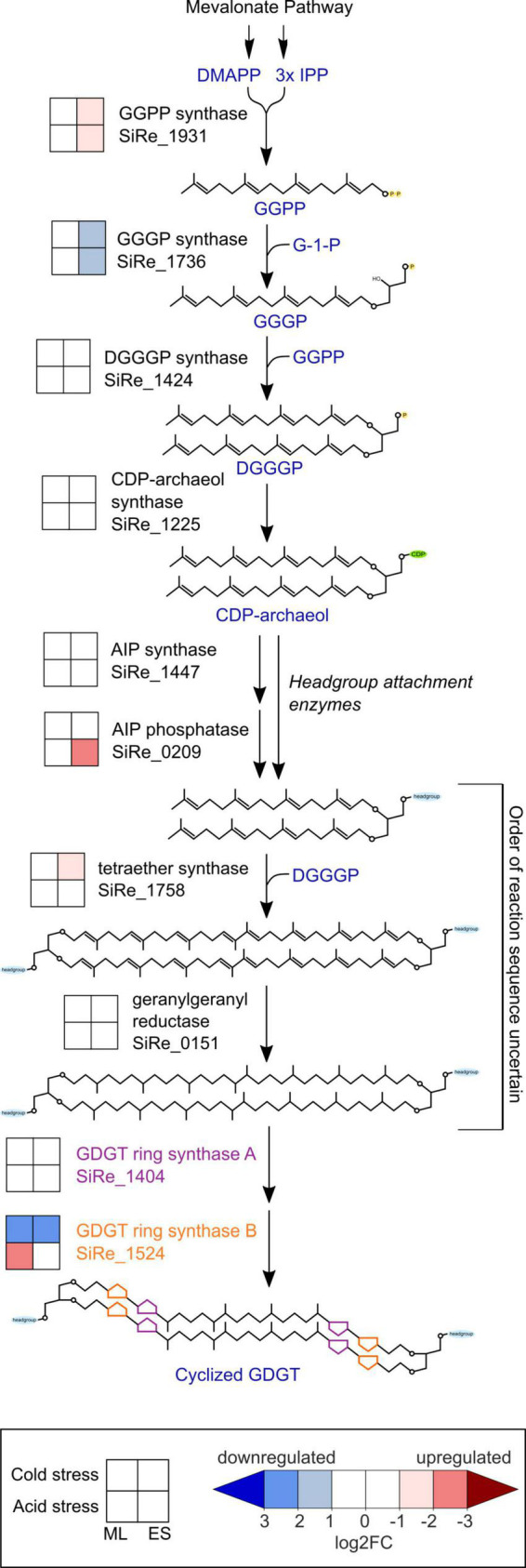
Transcriptomic changes within the GDGT biosynthesis pathway in response to cold stress or acid stress. GDGT, glycerol dibiphytanyl glycerol tetraether; DMAPP, dimethylallyl pyrophosphate; IPP, isopentenyl pyrophosphate; GGPP, geranylgeranyl diphosphate; GGGP, 3-O-geranylgeranyl-sn-glyceryl-1-phosphate; DGGGP, 2,3-bis-O-geranylgeranyl-sn-glyceryl-1-phosphate; CDP, cytidine diphosphate; AIP, archaetidylinositol phosphate; ML, mid-logarithmic phase; ES, early stationary phase.

To better understand the mechanisms by which GDGT cyclization is affected by environmental stresses, we looked for differential expression among GDGT biosynthesis genes (Figure 5), particularly the GDGT ring synthase genes *grsA* and *grsB* that are essential for the formation of cyclic moieties on GDGTs (Zeng et al., 2019). *S. islandicus* did not differentially express *grsA* (SiRe_1404) under any condition (| log2FC| always <1) while *grsB* (SiRe_1524) expression responded to both acid and cold stress. *S. islandicus* upregulated *grsB* at mid-log phase under acid stress conditions (log2FC = −2.1) and downregulated *grsB* in both growth phases under cold stress (log2FC = 2.1 and 2.5 at mid-log and early stationary phase, respectively).

Unique to cold stress conditions during the early stationary phase, *S. islandicus* upregulated its homolog of tetraether synthase (*tes*; SiRe_1758). Tetraether synthase catalyzes the condensation of pairs of archaeol to form GDGTs (Lloyd et al., 2022; Zeng et al., 2022), and the upregulation of *tes* observed here may indicate increased tetraether production induced by cold stress.

Several other GDGT biosynthesis genes showed differential expression under both stress conditions. Two genes encoding enzymes that catalyze consecutive steps in the formation of GDGT synthesis intermediate DGGGP displayed transcriptional shifts under both acid and cold stress conditions during early stationary phase: GGPP synthase (SiRe_1931) was upregulated while GGGP synthase (SiRe_1736) was downregulated (Figure 5). While GGPP and GGGP are both GDGT precursors, we note that both also serve as precursors for dialkyl glycerol diether (DGD) lipid synthesis, and GGPP serves as a precursor for other polyterpene compounds including quinones and polyprenols (Koga and Morii, 2007; Salvador-Castell et al., 2019).

*Saccharolobus islandicus* upregulated one headgroup attachment related gene, the putative archaetidylinositol phosphate (AIP) phosphatase (SiRe_0209), under acid stress conditions during early stationary phase. AIP phosphatase may dephosphorylate archaetidylinositol AIP to form the archaetidylinositol (AI) headgroup (Morii et al., 2009). However, AIP synthase (SiRe_1447), which catalyzes the initial attachment of 1L-myo-inositol-1-phosphate (IP) headgroup to CDP-archaeol to form AIP, was not differentially expressed under either stress condition (Morii et al., 2009). Calditol synthase (*cds*), for calditol headgroup synthesis, was previously shown to be important for acid tolerance in *S. acidocaldarius* (Zeng et al., 2018). Despite this, the *S. islandicus* homolog of *cds* (SiRe_1626) was not a DEG in our dataset, suggesting that it may be constitutively expressed rather than transcriptionally regulated by acid stress conditions (Zeng et al., 2018). While *Sulfolobales* sp. lipids are known to have other possible headgroups besides IP and calditol, the genes for synthesizing these lipid headgroups are yet to be identified (Langworthy, 1977; De Rosa et al., 1980b; Daiyasu et al., 2005; Jensen S. et al., 2015; Jensen S. M. et al., 2015).

We also looked for DEGs related to the mevalonate (MVA) pathway, which forms isoprenoid building blocks as precursors for GDGT biosynthesis. We found five MVA pathway genes to be DEGs, all upregulated during early stationary phase. Both acid and temperature stress induced upregulation of the genes encoding for the first three steps of the MVA pathway: acetoacetyl-CoA thiolase (AACT; SiRe_1461), 3-hydroxy-3-methylglutaryl-CoA synthase (HMGS; SiRe_1462), and 3-hydroxy-3-methylglutaryl-CoA reductase (HMGR; SiRe_1459) (Supplementary Figure 1). The final two MVA pathway-related DEGs were phosphomevalonate kinase (PMK; SiRe_2253), upregulated only under cold stress, and isopentenyl phosphate kinase (IPK; SiRe_1929), upregulated only under acid stress. PMK is a part of the classical MVA pathway and is absent in many archaea, but present in the *Sulfolobales* (Jain et al., 2014). IPK is part of the “modified” or “alternative” MVA pathway that is conserved among archaea (Jain et al., 2014). The observed PMK and IPK differential expression results suggest that their respective MVA pathways are favored under specific stress conditions.

#### 3.3.2. Common transcriptional responses to both acid and cold stress

We analyzed the *S. islandicus* response to acid or cold stress across the entire transcriptome. Both environmental stressors, a pH unit shift in acidity (from pH 3.4 to 2.4) or a ten degree decrease in temperature (from 76 to 66°C), induced 467 common DEGs with the same directionality of expression change at early stationary phase (Figure 4B). We highlight several of these common DEGs below and suggest that they may provide candidate genes for studying generalized stress response in *S. islandicus* facing other types of environmental perturbations.

**FIGURE 6:**
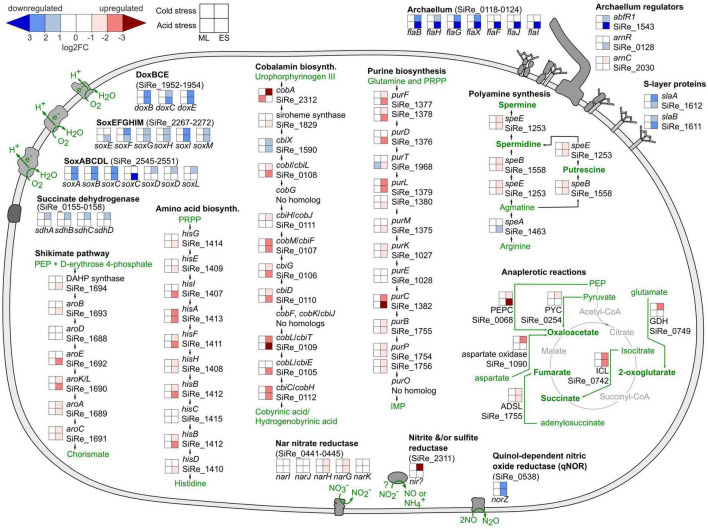
*Saccharolobus islandicus* cell biology summary highlighting common acid and cold stress transcriptional responses. Displayed operons or pathways show differential expression induced by each stress relative to optimal growth conditions as the control. PEP, phosphoenolpyruvate; PRPP, phosphoribosyl pyrophosphate; DAHP, 3-deoxy-D-arabinoheptulosonate-7-phosphate synthase; IMP, inosine monophosphate; PEPC, phosphoenolpyruvate carboxylase; PYC, pyruvate carboxylase; ADSL, adenylosuccinate lyase; ICL, isocitrate lyase; GDH, glutamate dehydrogenase; ML, mid-logarithmic phase; ES, early stationary phase.

Differential gene expression results supported decreased energy production and motility as part of the acid and cold stress response in *S. islandicus*. At early stationary phase under both stresses, *S. islandicus* downregulated all three terminal oxidases (DoxBCE, SoxABCDL, and SoxEFGHIM) of its electron transport chain (ETC) (Figure 6). Succinate dehydrogenase, which reduces quinones upstream of cytochromes and terminal oxidases in the ETC, was downregulated in response to cold stress. Decreased transcription of ETC genes suggests decreased or slower energy production that is consistent with the slower growth rates observed in response to both acid and cold stress. Similarly, differential expression of archaellum genes and archaellum regulators suggested that *S. islandicus* decreased motility in response to both stressors. Most or all of the *S. islandicus* archaellum-encoding *fla* cluster showed downregulation in response to both stresses during early stationary phase (Figure 6). Additionally, differential expression of archaellum regulators *abfR1*, *arnR*, and *arnC* in response to acid and/or cold stress all supported repression of the archaellum component *flaB* (Figure 6; Lassak et al., 2013; Orell et al., 2013; Hoffmann et al., 2017). The *slaA* and *slaB* genes encoding the *S. islandicus* S-layer, which anchors the archaellum, were also downregulated in response to acid and cold stress during later growth (Figure 6; Banerjee et al., 2015; Tsai et al., 2020). Interestingly, two hypothetical genes (SiRe_0125 and SiRe_0126) located between the *S. islandicus fla* cluster and *arnR* were also downregulated during early stationary phase under both stresses. These genes do not have homologs in *S. acidocaldarius*, the organism used for the majority of archaellum characterization work to date (Albers and Jarrell, 2015), but the genomic context and consistent expression pattern of these genes in *S. islandicus* suggest they contribute to *S. islandicus* archaellum-related motility.

While *S. islandicus* seemed to decrease energy production and motility, differential expression results indicated upregulation of other functions in response to acid and cold stressors. For example, upregulation of homologous recombination repair (HRR) genes encoding for the Holliday junction cleavage enzyme (SiRe_1431) and the Hel308A/Hjm DNA repair helicase (SiRe_0250) suggest increased DNA repair in acid and cold stress conditions during early stationary phase (Komori et al., 1999; Li et al., 2008). Similarly, *S. islandicus* upregulated numerous RNA polymerase genes, ribosomal protein genes, and translation initiation factors suggesting increased transcription and translation under both stressors. Differential expression, largely downregulation, of numerous genes encoding VapBC toxin-antitoxin system proteins (Supplementary Table 5), also supports changes in translation activity as part of the *S. islandicus* acid and cold stress responses. VapBC toxin-antitoxin systems affect the translation of specific RNAs (Walling and Butler, 2019), and have previously been associated with archaeal heat stress and uranium stress responses (Cooper et al., 2009, 2023; Maezato et al., 2011; Mukherjee et al., 2017).

In addition to DEGs supporting upregulated DNA repair, transcription, and translation, our data also suggested increased anabolic activity in response to acid and cold stress during early stationary phase. *S. islandicus* upregulated pathways for biosynthesis of the vitamin and cofactor cobalamin, the purine nucleotide precursor inosine 5′-monophosphate (IMP), the amino acid histidine, the versatile biochemical intermediate chorismate, and polyamines (Figure 6). *S. islandicus* concurrently increased its expression of anaplerotic enzyme-encoding genes (Figure 6), which replenish TCA cycle intermediates that are diverted for biosynthesis. Thus, the evidence for increased anaplerosis complements the evidence for increased biosynthesis in response to acid and cold stressors.

Interestingly, several nitrate reduction genes shifted in response to acid and cold stress. *S. islandicus* REY15A belongs to a subset of *S. islandicus* strains with nitrate reduction genes predicted in their genomes (Guo et al., 2011). During early stationary phase of both stress conditions, *S. islandicus* downregulated a qNOR nitric oxide reductase gene (*norZ*; SiRe_0538) and upregulated the catalytic and electron transfer subunits of the Nar nitrate reductase (*narG*; SiRe_0444 and *narH*; SiRe_0443, respectively) (Figure 6). qNORs use electrons derived from the quinone pool to reduce nitric oxide as part of denitrification or as a means of scavenging and detoxifying nitric oxide (Hendriks et al., 2000). Nar is a nitrate-reducing system typically associated with anaerobic nitrate respiration and generating proton motive force. An annotated sulfite reductase (SiRe_2311), which could be bifunctional for sulfite and nitrite, was also upregulated in cold stress conditions (Crane and Getzoff, 1996). Although anaerobic nitrate respiration by *S. islandicus* is not expected, particularly in our aerobic experiments, the differential expression of these dissimilatory nitrate reduction genes suggest they may play a role in the acid and cold stress responses of *S. islandicus*.

Finally, given the slower growth rates induced by acid and cold stress (Figure 2), we were interested in analyzing the regulation of the *S. islandicus* cell division (*cdv*) genes. Unfortunately, mean normalized counts for *cdvA*, *cdvB*, and *cdvC* (SiRe_1173-1175) were all lower than the optimal threshold for differential gene expression analysis, and we could not determine whether they were differentially transcribed in response to either stressor (Supplementary Table 4).

#### 3.3.3. Transcriptional responses unique to acid stress

Acid stress yielded strong shifts in the *S. islandicus* transcriptome. The number of acid-specific DEGs outnumbered both the common and cold-specific DEGs at each sampled growth phase, and early stationary phase induced more acid-specific DEGs than mid-log phase (Figure 4). These DEGs provide insight into the specific stress response of thermoacidophilic archaea to downshifts in environmental pH, which are common in their native hot springs. We detail the acid-specific DEGs here.

In response to acid stress conditions, *S. islandicus* upregulated a membrane bound A-type ATPase that may function as a proton pump. Previous work demonstrated ATP-hydrolyzing activity by partial complexes of A-type ATPase purified from *Sulfolobales* (Konishi et al., 1987; Lübben and Schäfer, 1987; Hochstein and Stan-Lotter, 1992), and the *atpK* sequence suggests translocation of H^+^ rather than Na^+^ (Grüber et al., 2014). Based on these lines of evidence, we suggest that the observed upregulation of six of eight subunits of the A-type ATPase (SiRe_1437-1442; log2FC range: −1.3 to −1.1) during early stationary phase may indicate ATP-fueled proton-pumping in response to acidic conditions (Supplementary Figure 2).

Acid stress conditions also caused *S. islandicus* to upregulate biosynthesis pathways for amino acids including arginine, lysine, and tryptophan (Supplementary Figure 3). Interestingly, the upregulated arginine and tryptophan pathways directly relate to several of the biosynthetic pathways that *S. islandicus* upregulated under both acid and cold stress (Figure 6). For example, biosynthesized arginine serves as the precursor for the polyamine synthesis pathway, and tryptophan biosynthesis relies on the shikimate pathway’s product, chorismate.

Acid-specific differential expression results indicated upregulation of genes with repair and turnover functions, suggesting that acidic conditions likely damaged *S. islandicus* DNA, RNA, and proteins. During early stationary phase, *S. islandicus* upregulated DNA damage sensing and repair genes including two HRR genes, *mre11* (SiRe_0063; log2FC = −1.0) and *rad50* (SiRe_0062; log2FC = −1.3), DEAD/DEAH box helicase domain genes (SiRe_1605 and SiRe_1977; log2FC = −1.9 and −1.8, respectively), and the thermophile-specific reverse gyrase, *topR1* (SiRe_1581; log2FC = −1.6) (Forterre, 2002; Kampmann and Stock, 2004; Fröls et al., 2007; Quaiser et al., 2008; Song et al., 2016). The thermosome (subunits α, SiRe_1214; β, SiRe_1716; γ, SiRe_2245), a chaperonin with roles in protein synthesis and repair, was also upregulated throughout growth (log2FC range: −2.1 to −1.3) under acid stress conditions (Klumpp and Baumeister, 1998). With regard to molecular degradation processes, we observed upregulation of the exosome complex (SiRe_1273-1275; log2FC range: −1.3 to −1.0), which mediates polyadenylation and RNA degradation (Portnoy et al., 2005), and upregulation of the PAC2 assembly factor (*pbaA*; SiRe_1724; log2FC = −1.1) for the protein-degrading 20S proteasome (Kusmierczyk et al., 2011).

Consistent with the observations of DEGs related to molecular turnover processes, *S. islandicus* may have also increased fatty acid degradation as an acid-specific response. Six genes belonging to a genomic cluster of fatty acid metabolism genes were upregulated under acid stress during mid-log and/or early stationary phase (Supplementary Figure 4). Accordingly, the fatty acid metabolism repressor, TetR (SiRe_0301), co-localized within this cluster did not show differential expression (Wang et al., 2019). However, an adjacent Fis family transcriptional regulator (SiRe_0302) was upregulated under acid stress suggesting that it may play a role in regulating fatty acid metabolism of *S. islandicus*. Previous work linked fatty acid metabolism genes to acid stress adaptation in *Saccharolobus solfataricus* as a possible means of accelerating membrane turnover (McCarthy et al., 2016).

Downregulated DEGs specific to acid stress may represent genes and functions that do not play important roles in *S. islandicus* under low pH conditions. Downregulated acid-specific DEGs included 15 *orfB* elements belonging to the IS200/IS605 family of transposable elements, all of which showed downregulation (log2FC range: 1.3–4.4) at early stationary phase. We also observed acid-specific downregulation of one small heat shock protein chaperone (SiRe_2601; log2FC = 2.8) and two HtpX proteases (SiRe_0639 and SiRe_2013; log2FC = 1.7 for both) suggesting that these genes may not be important to the protein repair and turnover processes in acidic conditions. Finally, of the four universal stress proteins (USPs) predicted in the *S. islandicus* REY15A genome, only one was an upregulated acid-specific DEG (SiRe_1457; log2FC = −1.4) while all others were downregulated (SiRe_0634, SiRe_2404, and SiRe_2055), two of which were acid-specific responses (SiRe_0634 and SiRe_2402; log2FC = 2.2 and 1.6, respectively).

#### 3.3.4. Transcriptional responses unique to cold stress

Cold temperature stress induced unique transcriptional changes in *S. islandicus* that were not observed under acid stress. Although cold stress induced less than half the number of unique DEG responses compared to the number of acid-specific DEGs (Figure 4B), the majority of cold-specific DEGs occurred during early stationary phase rather than mid-log phase, consistent with observations of the acid-specific DEGs relative to growth phase (Figure 4). The DEGs unique to cold stress may provide insight into cold-specific responses of thermoacidophilic archaea, and we highlight several observations related to carbon and energy metabolism, transposases, and DNA repair. At early stationary phase, *S. islandicus* downregulated all four subunits comprising succinate dehydrogenase (SiRe_0155-0158; log2FC range: 1.2–1.3), which plays a role in both the TCA cycle and the ETC (Figure 6). Cold stress also induced upregulation of numerous predicted transposase-encoding genes at early stationary phase including 11 *orfB* elements (log2FC range: −3.4 to −1.3), 6 ISH3 family transposases (log2FC range: −3.6 to −2.7), and 1 ISH6 family transposase (log2FC = −3). Upregulation of transposases in cold stress conditions contrasts the general downregulation of transposases observed in acid stress conditions. With regard to DNA repair, *S. islandicus* strongly upregulated the HRR-related Holliday junction endonuclease (*hje*, SiRe_0930; log2FC = −4.2) during early stationary phase. This particular Holliday junction resolvase has been proposed to have a viral origin and is only found in certain archaea (Kvaratskhelia and White, 2000a,b; Middleton et al., 2004). Strong upregulation of *hje* may indicate that it is particularly important for cold-induced DNA damage.

### 3.4. Proteomic responses to acid and cold stress

**FIGURE 7:**
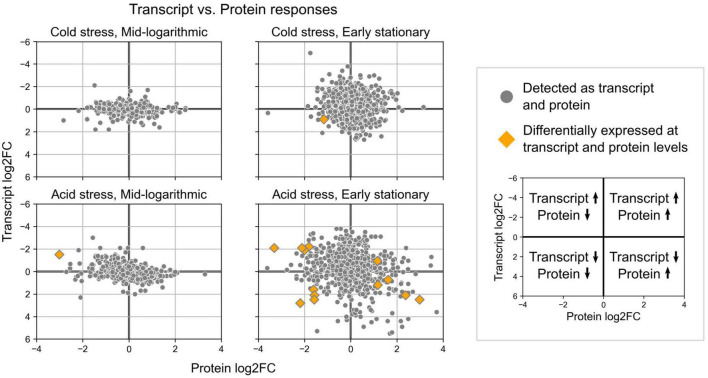
Expression change results from differential gene expression and differential protein abundance analyses for loci detected in both datasets. Orange diamonds highlight the few loci that showed significant changes in both transcript expression and protein abundances.

Quantitative proteomics revealed protein level changes associated with acid and cold stress responses of *S. islandicus*. We detected a total of 1,107 unique proteins, which account for 42% of the total predicted proteins (2,644) encoded by the *S. islandicus* REY15A genome. Differential abundance analysis revealed that 18 proteins significantly changed abundance levels under acid stress and 7 proteins significantly changed abundance under cold stress (Table 1). Of all the observed significant protein abundance changes, only six showed consistent transcriptomic changes at corresponding growth phase and stress condition including the heat shock protein (SiRe_2601) downregulated at early stationary phase under acid stress (Table 1). Another nine significant protein changes showed transcriptional changes at corresponding growth phase and stress condition, but with opposite directionality (Table 1 and Figure 7).

**TABLE 1:** Summary of *S. islandicus* proteomic responses to tested environmental stresses.

Condition and phase	Log2FC*	Annotation	Locus tag	Transcript change direction
**Acid stress**				
Mid-log	2.08	Ornithine carbamoyltransferase	SiRe_1208	N/A
	−3.01	Conserved hypothetical protein	SiRe_2064	↑
	−2.29	Ketol-acid reductoisomerase	SiRe_0753	N/A
Early stationary	2.98	UDP-sulfoquinovose synthase, Agl3	SiRe_2623	↓
	2.38	AMP-dependent synthetase and ligase	SiRe_2451	↓
	1.94	Mandelate racemase (MR) subfamily	SiRe_2246	N/A
	1.62	Aminotransferase class V	SiRe_0032	↓
	1.17	Conserved hypothetical protein	SiRe_2599	↓
	1.15	Conserved hypothetical protein	SiRe_2391	↑
	−3.31	Formate dehydrogenase, alpha subunit	SiRe_2464	↑
	−2.58	Thioredoxin/glutaredoxin-like protein	SiRe_1684	N/A
	−2.19	Heat shock protein (Hsp20)	SiRe_2601	↓
	−2.11	Thermosome	SiRe_1716	↑
	−2.01	Thermosome	SiRe_1214	↑
	−1.81	Deoxyhypusine synthase	SiRe_1119	↑
	−1.70	Glutamine synthetase, type I	SiRe_1633	N/A
	−1.61	CoA-binding domain protein	SiRe_0989	↓
	−1.59	Conserved hypothetical protein	SiRe_2064	↓
	−1.57	Extracellular solute-binding protein family 1	SiRe_1066	↓
**Cold stress**				
Mid-log	1.22	A-type ATPase subunit, AtpI	SiRe_1444	N/A
	−2.83	Arginosuccinate lyase	SiRe_1371	N/A
	−2.19	Ketol-acid reductoisomerase	SiRe_0753	N/A
	−1.44	Glycerol kinase	SiRe_0511	N/A
	−1.29	Beta-glucosidase	SiRe_2202	N/A
	−1.08	Amidohydrolase	SiRe_0643	N/A
Early stationary	1.85	Short-chain dehydrogenase/reductase SDR	SiRe_0099	N/A
	−1.17	Oxidoreductase molybdopterin binding protein	SiRe_2037	↓

*Negative log2FC values indicate decreased protein levels under stress conditions relative to optimal conditions. Positive log2FC values correspond with increased protein levels under the stress condition. Log2FC SE values can be found in Supplementary Table 6. If applicable, “Transcript Change Direction” column indicates the direction of expression change of corresponding transcripts.

Some of the largest magnitude protein level changes in *S. islandicus* included the downregulation of formate dehydrogenase alpha subunit (SiRe_2464; log2FC = −3.31) and the upregulation of UDP-sulfoquinovose synthase Agl3 (SiRe_2623; log2FC = 2.98), both under acidic stress conditions during early stationary phase. SiRe_2464 is homologous to the non-canonical formate dehydrogenase (FDH) YjgC subunit from *Bacillus subtilis* (*E*-value 0.0, 53% identities), which complexes with a second subunit to form a FDH that couples formate oxidation with menaquinone reduction (Arias-Cartin et al., 2022). Agl3 forms UDP-sulfoquinovose, a component of S-layer and archaellum N-glycans that was important for *S. acidocaldarius* growth under high salt conditions (Meyer et al., 2013). Cold stress resulted in a strongly decreased abundance of the arginine biosynthesis protein argininosuccinate lyase ArgH (SiRe_1371; log2FC = −2.83) during mid-log phase. Despite these proteins showing some of the highest magnitude changes in our proteomic dataset, none had consistent transcriptional changes at corresponding growth phase and stress conditions, suggesting *S. islandicus* might engage significant post-transcriptional regulation in its acid and cold stress responses.

## 4. Discussion

To better understand the cell biology of GDGT-forming archaea when they modify their membranes, we quantified the lipid, protein, and transcript responses of *S. islandicus* cultivated under acid or cold stress. Only one study to-date has used proteomics and transcriptomics in parallel with membrane lipid profiles to characterize a thermoacidophilic archaeon’s stress response, specifically the *S. acidocaldarius* nutrient limitation response (Bischof et al., 2019). We subjected the model thermoacidophile, *S. islandicus* REY15A, to acid or cold stress, inducing shifts in GDGT cyclization (Figure 3), protein expression (Table 1), and gene transcription (Figure 4). The RNA and protein data overlapped minimally, consistent with prior studies in another thermoacidophilic crenarchaeon, *S. acidocaldarius*, suggesting that stress responses in *Sulfolobales* sp. involve multiple levels of gene transcription and translation (Figure 7; Bischof et al., 2019; Benninghoff et al., 2021). Here, stress conditions induced hundreds of transcriptional changes and only tens of protein level changes in *S. islandicus*. While most of the differentially expressed proteins in *S. islandicus* under acid stress were also differentially transcribed, many of these loci showed opposite directionality between data type (Table 1). Overall, we focus this discussion first on GDGT profiles and expression responses of the GDGT ring synthases, then on the broader transcription responses of *S. islandicus* under acid or cold stress conditions.

### 4.1. Acid or cold stress effects on GDGTs and GDGT biosynthesis genes

As expected, acid and cold stress both induced shifts in the average cyclization of *S. islandicus* GDGTs. While each stress caused changes to the relative abundances of different individual GDGTs, both sets of stress-induced changes resulted in lower RI values indicating lower average cyclization (Figure 3). The GDGT responses of various archaeal groups to a wide variety of stressors have been reviewed in the literature (e.g., Oger and Cario, 2013; Law et al., 2020; Rastädter et al., 2020), but we limit our discussion here to comparisons between our data and GDGT studies examining similar stress conditions. Lower RI at lower temperature is consistent with previous laboratory studies of thermoacidophiles as well as mesophilic neutrophiles (Figure 3; De Rosa et al., 1980a; Uda et al., 2001, 2004; Boyd et al., 2011; Elling et al., 2015; Jensen S. et al., 2015; Feyhl-Buska et al., 2016; Cobban et al., 2020).

Shifts in archaeal GDGT cyclization in response to pH changes are more complex. We observed lower RI at lower pH in *S. islandicus* (Figure 3), which is consistent with previous laboratory experiments with *S. acidocaldarius* (Cobban et al., 2020). However, a coherent empirical relationship between archaeal GDGT cyclization and pH has not yet been established (Siliakus et al., 2017). Moreover, comparison of our data to prior reports of GDGT shifts in response to pH is difficult given that several other studies only examined effects of higher pH’s rather than lower pH (e.g., Shimada et al., 2008; Boyd et al., 2011). Thermoacidophilic archaea such as *S. islandicus* maintain circumneutral intracellular pH while living in extremely acidic environments, resulting in steep proton gradients across their membranes. Shifting GDGT cyclization serves as a pH homeostasis mechanism for thermoacidophiles to maintain these proton gradients (Baker-Austin and Dopson, 2007; Slonczewski et al., 2009). Highly cyclized GDGTs form more tightly packed and less permeable membranes that may help limit passive proton diffusion and in turn protect against cytoplasmic acidification (Gabriel and Chong, 2000; Shinoda et al., 2005; Baker-Austin and Dopson, 2007). Thus, we initially predicted *S. islandicus* might increase RI as one means of counteracting the higher external concentration of protons at lower pH. In contrast, *S. islandicus* exhibited lower RI at more acidic pH, indicating that its membrane was more permeable. If increased membrane permeability allowed greater proton diffusion into the cell, the stunted maximum cell density of our cultures grown under acid stress (Figure 2) could represent cells succumbing to cytoplasmic acidification. Other factors can affect archaeal membrane permeability including GDGT headgroup composition and it follows that quantitative headgroup analysis and/or direct cytoplasmic pH measurements would help validate our proposed hypothesis (Oger and Cario, 2013).

Under both stress conditions, *S. islandicus* GDGT compositions differed by growth phase. RI increased at early stationary phase relative to mid-log phase within both the acid and cold stress conditions, as well as in optimal growth conditions (Figure 3). This is consistent with previous observations from other archaeal batch studies sampled at similar growth phases including *S. islandicus* and *S. acidocaldarius* as well as the with *Picrophilus torridus* and *Nitrosopumilus maritimus* (Elling et al., 2014; Jensen S. et al., 2015; Feyhl-Buska et al., 2016; Cobban et al., 2020). We suggest that our observations from *S. islandicus* are also consistent with the findings from continuous culture experiments of *S. acidocaldarius* and *N. maritimus* showing that RI increases as electron availability decreases (Hurley et al., 2016; Quehenberger et al., 2020; Zhou et al., 2020). As our batch cultures grew, *S. islandicus* actively consumed medium nutrients and thereby depleted its available energy supply. Here we observed increased RI values from mid-log to early stationary phase, which we infer as a transition to more energy limited conditions.

**FIGURE 8:**
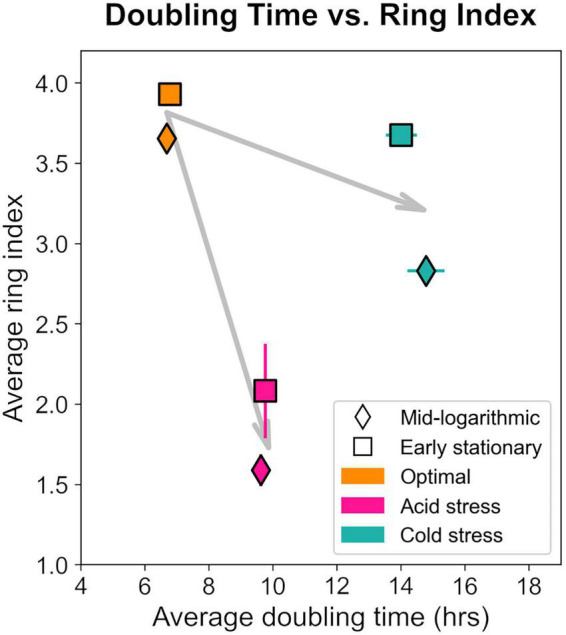
Relationship between ring index and doubling time from each growth condition and at each time point harvested. Each data point represents the average of triplicates and error bars represent standard error for both axes. Gray arrows represent the shift relative to optimum (orange) for acid (pink) or cold (teal) stresses.

Notably, *S. islandicus* RI and doubling time did not linearly covary when comparing data from all tested growth conditions suggesting that RI changes are not a result of growth rate alone (Figure 8). pH and temperature are distinct physical parameters that likely affect cell physiology and thereby GDGT composition differently. If we consider the general effects of acid stress and cold stress independently, it is clear that both stressors induced slower growth and decreased RI relative to optimal conditions. These observations are consistent with the broad trend of environmental stress causing longer doubling times and decreased RI as observed in *S. acidocaldarius* (Cobban et al., 2020).

To provide context for the shifts in *S. islandicus* GDGT compositions induced by acid and cold stress, we examined expression changes of known GDGT biosynthesis genes under each condition. Most notable were the expression changes in the *S. islandicus* GDGT ring synthase genes, *grsA* and *grsB*. The *grs* genes encode the GrsA and GrsB enzymes that are required for cyclopentane ring formation in *S. acidocaldarius* and *S. solfataricus* (Zeng et al., 2019). GrsA produces GDGTs 1 through 4 and GrsB subsequently acts on GDGT-4 to produce GDGTs 5 through 8 (Zeng et al., 2019). A recent study using RT-qPCR and Western blot analysis examined GDGT ring synthase expression in *S. acidocaldarius* cultured at more acidic pH or colder temperature than its optimal conditions of pH 3.5 and 75°C (Yang et al., 2023). Acid stress (pH 2.5) induced increased *grsB* and GrsB expression in *S. acidocaldarius* while cold stress (65°C) caused decreased *grsB* expression and undetectable levels of GrsB (Yang et al., 2023). In contrast, *S. acidocaldarius* did not show differential expression of *grsA* nor GrsA in either stress condition at 48 or 72 h timepoints, which are the closest analogs to the mid-log and early stationary phase timepoints in our experiments (Yang et al., 2023). Results from our study indicated similar expression patterns of the *grs* genes in *S. islandicus* grown under acid stress or cold stress. *S. islandicus* did not differentially express *grsA* or GrsA in response to either stress, supporting constitutive transcription of *grsA* and insignificant GrsA protein level changes (Figure 5). However, *S. islandicus* showed differential expression of *grsB* as responses to both stress conditions in alignment with observations from *S. acidocaldarius* (Figure 5; Yang et al., 2023). Under cold stress at both mid-log and early stationary phases, *S. islandicus* downregulated *grsB*, and consistent with the gene’s expected function, cold stress lipid profiles had lower abundances of GDGTs with ≥5 rings (Figure 3). Under acid stress, *S. islandicus* upregulated *grsB* at mid-log phase, but changes in acid stress lipid profiles did not correspond as predicted by GrsB function. That is, no GDGTs with ≥5 rings significantly increased under acid stress (Figure 3). The isomer of GDGT-5 (GDGT-5′) increased under acid stress, but did not result in an overall increased abundance of GDGTs with 5 rings. The inconsistency between increased *grsB* transcription and the predicted changes in acid stress GDGT profiles may be related to the fact that like most of the DEGs in this study, the abundance of GrsB protein did not increase in tandem with higher transcription. Additionally, GrsB requires GDGT-4 as a substrate to produce more cyclized GDGTs, and GDGT-4 abundance was starkly lower in *S. islandicus* acid stress lipid profiles relative to optimal conditions (Figure 3; Zeng et al., 2019). Finally, GDGT profiles are likely not only affected by the GDGT ring synthase transcription and protein levels quantified here, but also by variable Grs enzyme activity under different temperature or pH conditions and by potential reworking of GDGT cyclization by as yet unidentified enzymes. Overall, acid and cold stress both affected the regulation of *grsB* in *S. islandicus*, but only cold stress conditions resulted in differential *grsB* expression and GDGT-level changes consistent with GrsB function. Our results suggest that transcriptional data may be helpful for predicting GDGT-level changes in cold stress conditions, but do not reflect Grs enzyme activity under all stress conditions.

Differential gene expression of other GDGT biosynthesis genes suggested that *S. islandicus* may have adapted its membrane to acid and cold stress by means other than GDGT cyclization. For example, the polar headgroups attached to membrane lipids can play a role in controlling membrane permeability (Shimada et al., 2008; Oger and Cario, 2013). Although we did not quantify headgroups in our experiments and despite only a few known headgroup synthesis genes, our RNA-seq data indicated that *S. islandicus* upregulated AIP phosphatase (SiRe_0209) under acid stress, suggesting that the proportion of archaetidylinositol headgroups may have increased in response to acidic conditions. We also observed differential expression of two sequential GDGT biosynthesis genes in response to acid and cold stress, though the directionality of their expression changes differed. *S. islandicus* upregulated GGPP synthase (SiRe_1931) and downregulated GGGP synthase (SiRe_1736) (Figure 5). The downregulation of GGGP synthase and the lack of differential expression changes of numerous other GDGT synthesis genes (Figure 5) suggest that *S. islandicus* did not increase overall core lipid synthesis in response to acid or cold stress. However, the upregulation of GGPP synthase suggests increased production of GGPP, which is an intermediate for the syntheses of multiple polyterpenes beyond just core lipids. A potentially increased supply of GGPP could have been funneled toward the synthesis of polyterpenes like quinones and polyprenols, which have been proposed as archaeal membrane regulators for environmental stress response (Salvador-Castell et al., 2019). Broader characterizations of archaeal membrane components in response to environmental stressors could help shed light on these other adaptative mechanisms.

### 4.2. Broader transcriptional responses to acid or cold stress

RNA-seq enabled a comprehensive overview of *S. islandicus’* acid and cold stress responses beyond just lipid biosynthesis. Overall, *S. islandicus* differentially expressed many more genes during early stationary phase than mid-log phase under both stresses (Figure 4), possibly related to prolonged exposure to a given stressor and/or onset of growth limitation. These observations show the value in analyzing stress-induced expression changes at multiple growth phases and at later growth phases in particular. We also found that both acid and cold stress induced many of the same transcriptional responses in *S. islandicus*, which we propose to represent candidate general stress response genes that can be examined in future studies testing different environmental stressors. One common transcriptional response to both acid and cold stress included downregulation of ETC terminal oxidases, suggesting decreased oxidative phosphorylation and thereby a reduction in ATP generation and proton pumping. The drop in ATP supply would stymie energy-requiring processes, which is consistent with the slower growth rates we observed under both stress conditions (Figure 2). In addition to a slowed growth rate, acid stress also caused a significant decrease in maximum cell density not seen in cold stress conditions (Figure 2). Decreased growth yield suggests that *S. islandicus* acid stress response was energetically costly and/or that *S. islandicu*s could not successfully counteract the negative effects of acid stress.

Cell motility also decreased in response to both stressors. Expression changes of the archaellum and related regulator genes indicated that *S. islandicus* decreased archaellum activity in response to both acid and cold stresses, contrasting the increased motility response by *S. acidocaldarius* to nutrient limitation (Szabó et al., 2007; Lassak et al., 2012; Haurat et al., 2017; Hoffmann et al., 2017; Bischof et al., 2019). This difference could indicate that unlike nutrient limitation, cells facing pH or temperature stress may conserve energy rather than swimming to find more favorable conditions.

*Saccharolobus islandicus* upregulated other cellular processes as common responses to acid and cold stress. Transcription and translation activities increased, based on upregulation of genes encoding for RNA polymerase, ribosomal proteins, and translation initiation factors. Differential expression of VapBC toxin-antitoxin systems also indicated changes in translation across our experimental conditions. Upregulated HRR genes and purine synthesis genes suggest increased DNA repair activities, and increased expression of anaplerotic reaction genes suggests increased biosynthetic activity. Accordingly, DEGs supported increased biosynthesis of polyamines, histidine, cobalamin, and chorismate in response to both stressors.

The upregulation of polyamine synthesis under both acid and cold stress was notable given that polyamines are commonly referenced as cytoplasmic buffers and thereby an acid-specific adaptation (e.g., Slonczewski et al., 2009; Lewis et al., 2021). While upregulation of polyamine synthesis genes may have ameliorated cytoplasmic acidification in acid stress conditions, the advantage of increased cytoplasmic buffering is less clear under cold stress. As such, we suggest that polyamines may have contributed to the acid and cold stress responses of *S. islandicus* by some other mechanism(s). For example, polyamines are known to play an important role in translation as they are essential for ribosome activity (Londei et al., 1986). Given that observed DEGs indicated increased translation in response to both acid and cold stress, we propose that *S. islandicus* could have increased polyamine synthesis to support translation activity. Overall, upregulated polyamine synthesis as response to both stressors suggest that polyamines may contribute to the *S. islandicus* acid and cold stress responses by multiple mechanisms.

Low pH induced a strong physiological response in *S. islandicus* relative to cold temperature. The number of DEGs, magnitude of RI changes, and magnitude of cell density impairment were all greater in response to acidic stress. Consistent with the damage to protein and nucleic acids that can result from cytoplasmic acidification (Lund et al., 2014), acid-specific DEGs showed that *S. islandicus* upregulated genes related to degradation and repair processes for molecules including proteins, nucleic acids, and fatty acids. One of the most notable acid-specific transcriptional responses involved upregulation of an A-type ATPase that we suggest functions as a proton pump based on evidence from enzyme purification and gene sequence studies (Konishi et al., 1987; Lübben and Schäfer, 1987; Hochstein and Stan-Lotter, 1992; Grüber et al., 2014). Proton pumping expels protons from the cytoplasm and is a common mechanism to combat pH stress (Baker-Austin and Dopson, 2007; Slonczewski et al., 2009). Terminal oxidases of the ETC also function as proton pumps, but *S. islandicus* downregulated all three of its terminal oxidases under acid stress suggesting a need for an alternate proton pump like the A-type ATPase. Altogether, the acid-specific DEGs such as the A-type ATPase provide insight to the ways that *S. islandicus* is affected by and responds to decreased pH.

The insertion sequence (IS) DEGs observed in our experiments notably showed opposite changes in expression between acid and cold stress conditions. Under both stressors, *S. islandicus* differentially expressed IS elements belonging to the IS200/605, ISH3, or ISH6 families of transposable elements, which are restricted to, or ancestral to, archaea (Filée et al., 2007). Acid stress induced consistent downregulation of these transposable elements whereas cold stress induced almost entirely upregulation shifts. These observations suggest that transposition frequency in *S. islandicus* may increase under cold stress and decrease under acid stress.

## 5. Conclusion

Our study characterizes *S. islandicus* GDGT lipid and expression level responses to acid stress and cold stress. Both stress conditions led to decreased GDGT cyclization and differential expression of *grsB*. Our observations of *grsB* downregulation and decreased GDGT cyclization in response to cold stress show that *S. islandicus* modifies its GDGT profile and GDGT ring synthase transcription consistently with cold temperature responses of closely related thermoacidophilic Crenarchaea (e.g., De Rosa et al., 1980a; Jensen S. et al., 2015; Cobban et al., 2020; Yang et al., 2023). Acid stress induced upregulation of *grsB* in *S. islandicus* also supports previous observations of pH affecting *grsB* transcription in *S. acidocaldarius* (Yang et al., 2023). However, our observations of decreased cyclization in acid stress lipid profiles, despite *grsB* upregulation, was counter to our predictions that *S. islandicus* might increase GDGT cyclization to reduce membrane permeability and limit passive proton diffusion. These results suggest that higher GDGT cyclization is not or perhaps cannot always be employed as an acid stress response strategy by acidophilic archaea. Based on transcriptional evidence, we suggest that *S. islandicus* may selectively allocate ATP consumption toward other acid stress response mechanisms aside from GDGT cyclization. Acid-stressed *S. islandicus* only upregulated *grsB* during mid-log phase while other acid response mechanisms such as proton pumping and molecular repair were actively upregulated during mid-log and/or early stationary phase. Decreased GDGT cyclization and increased transcription of alternate pH homeostasis responses indicate that membrane modification may not be the primary mechanism enabling *S. islandicu*s to survive acid stress. Together this work demonstrates how *S. islandicus* modifies its GDGTs and expression patterns in response to pH and temperature shifts. Future archaeal stress response studies using observations of both core and intact polar membrane lipid compositions, gene expression, and protein expression will help broaden our understanding of the intracellular factors that lead to archaeal membrane adaptations in response to environmental perturbations.

## Data availability statement

The datasets presented in this study can be found in online repositories. The names of the repository/repositories and accession number(s) can be found in the article/Supplementary material.

## Author contributions

BC and WL conceptualized and designed the experiments. BC performed the experiments and RNA and lipid extractions. LZ and JW performed the protein extractions, proteomics analysis, and proteomic data generation. FE and AP performed the lipid analysis and lipid data interpretation. BC and ÖM analyzed the expression data. BC and WL wrote the manuscript with contributions from JW and editorial expertise from EE. All authors contributed to the article and approved the submitted version.

## Footnotes

1
https://git.dartmouth.edu/leavitt_lab/cobban-saci-lipids-batch-and-fed-batch-2020

2
https://sourceforge.net/projects/bbmap/

3
github.com/waldbauerlab

4
massive.ucsd.edu
