# Supplementary material for: Membrane lipid and expression responses of Saccharolobus islandicus REY15A to acid and cold stress
Source: Front Microbiol. 2023 Aug 15;14:1219779. doi: 10.3389/fmicb.2023.1219779 (PMC10465181; doi:10.3389/fmicb.2023.1219779)
Supplement: Supplementary file 1 [file Presentation_1.pptx]

## Slide 1
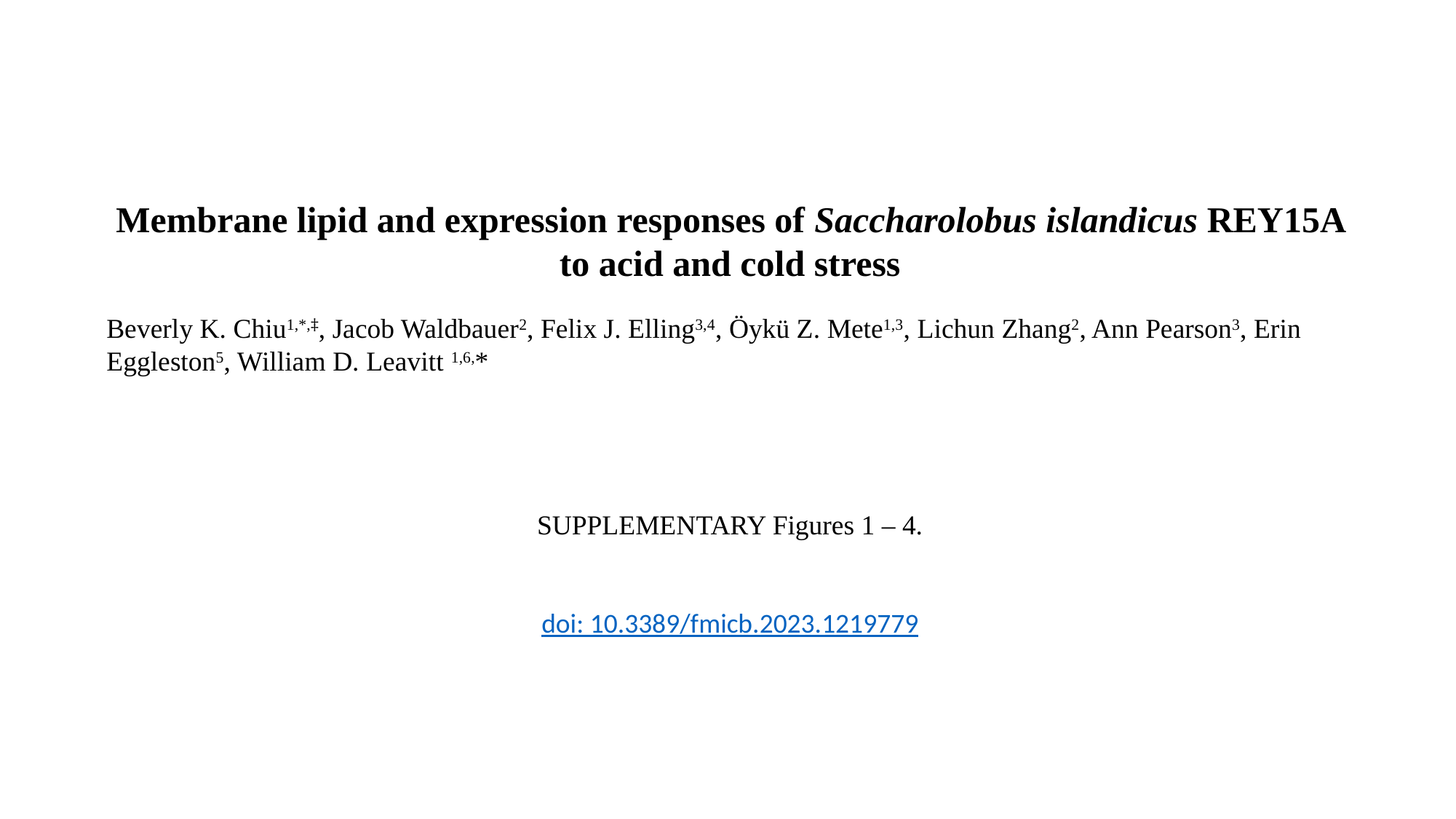

Membrane lipid and expression responses of Saccharolobus islandicus REY15A to acid and cold stress
Beverly K. Chiu1,*,‡, Jacob Waldbauer2, Felix J. Elling3,4, Öykü Z. Mete1,3, Lichun Zhang2, Ann Pearson3, Erin Eggleston5, William D. Leavitt 1,6,*
SUPPLEMENTARY Figures 1 – 4.
doi: 10.3389/fmicb.2023.1219779

## Slide 2
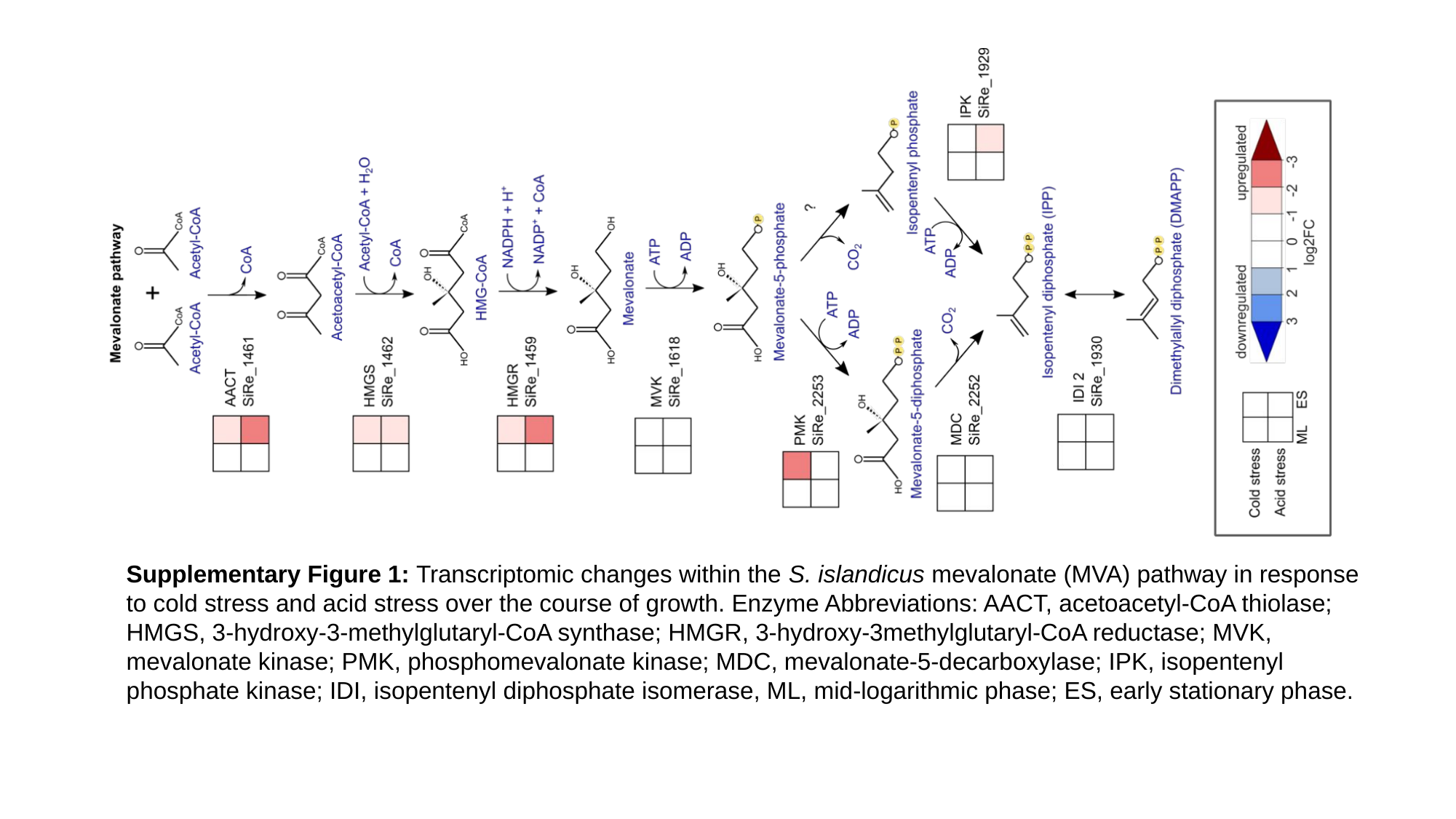

Supplementary Figure 1: Transcriptomic changes within the S. islandicus mevalonate (MVA) pathway in response to cold stress and acid stress over the course of growth. Enzyme Abbreviations: AACT, acetoacetyl-CoA thiolase; HMGS, 3-hydroxy-3-methylglutaryl-CoA synthase; HMGR, 3-hydroxy-3methylglutaryl-CoA reductase; MVK, mevalonate kinase; PMK, phosphomevalonate kinase; MDC, mevalonate-5-decarboxylase; IPK, isopentenyl phosphate kinase; IDI, isopentenyl diphosphate isomerase, ML, mid-logarithmic phase; ES, early stationary phase.

## Slide 3
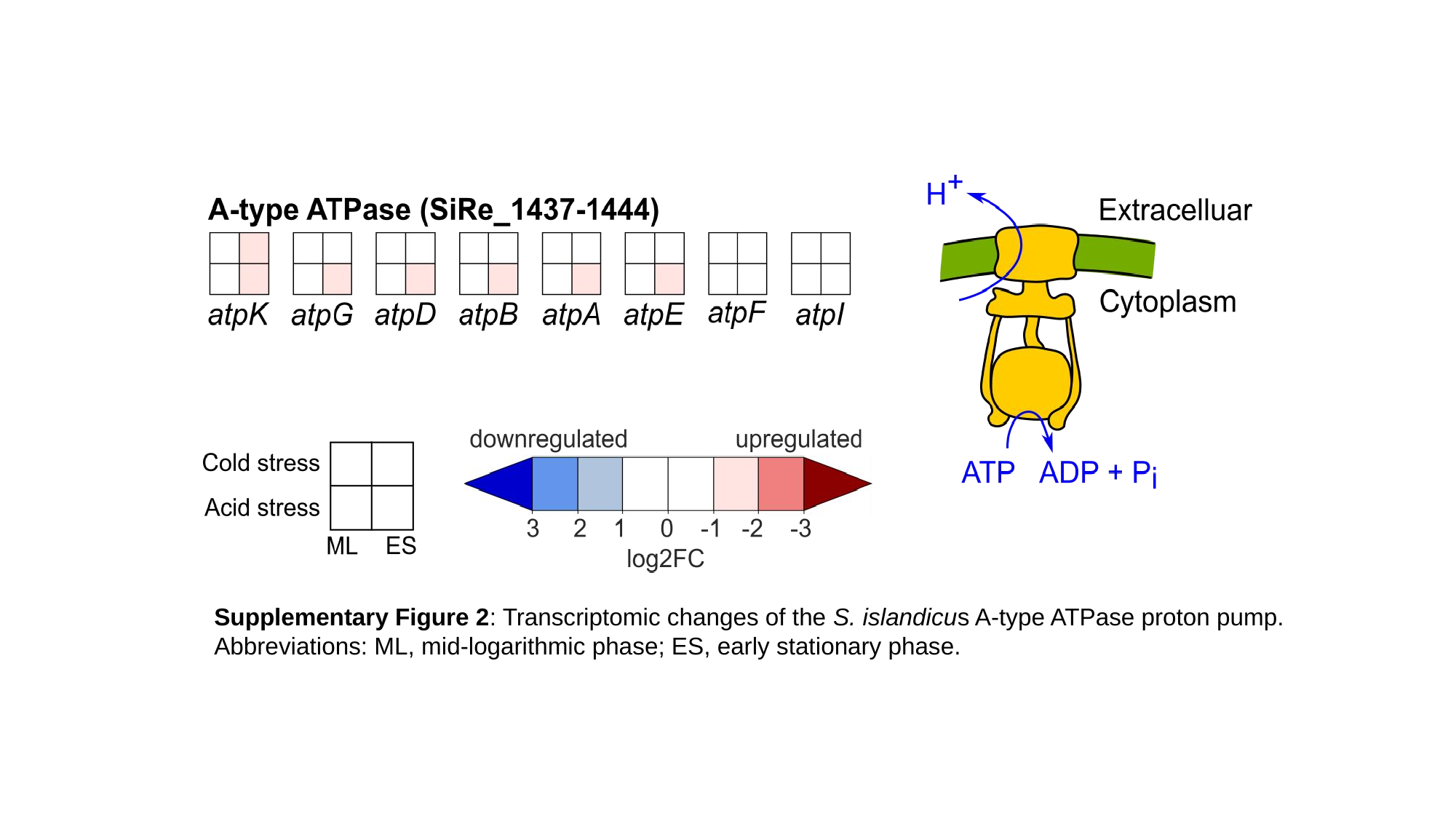

Supplementary Figure 2: Transcriptomic changes of the S. islandicus A-type ATPase proton pump. Abbreviations: ML, mid-logarithmic phase; ES, early stationary phase.

## Slide 4
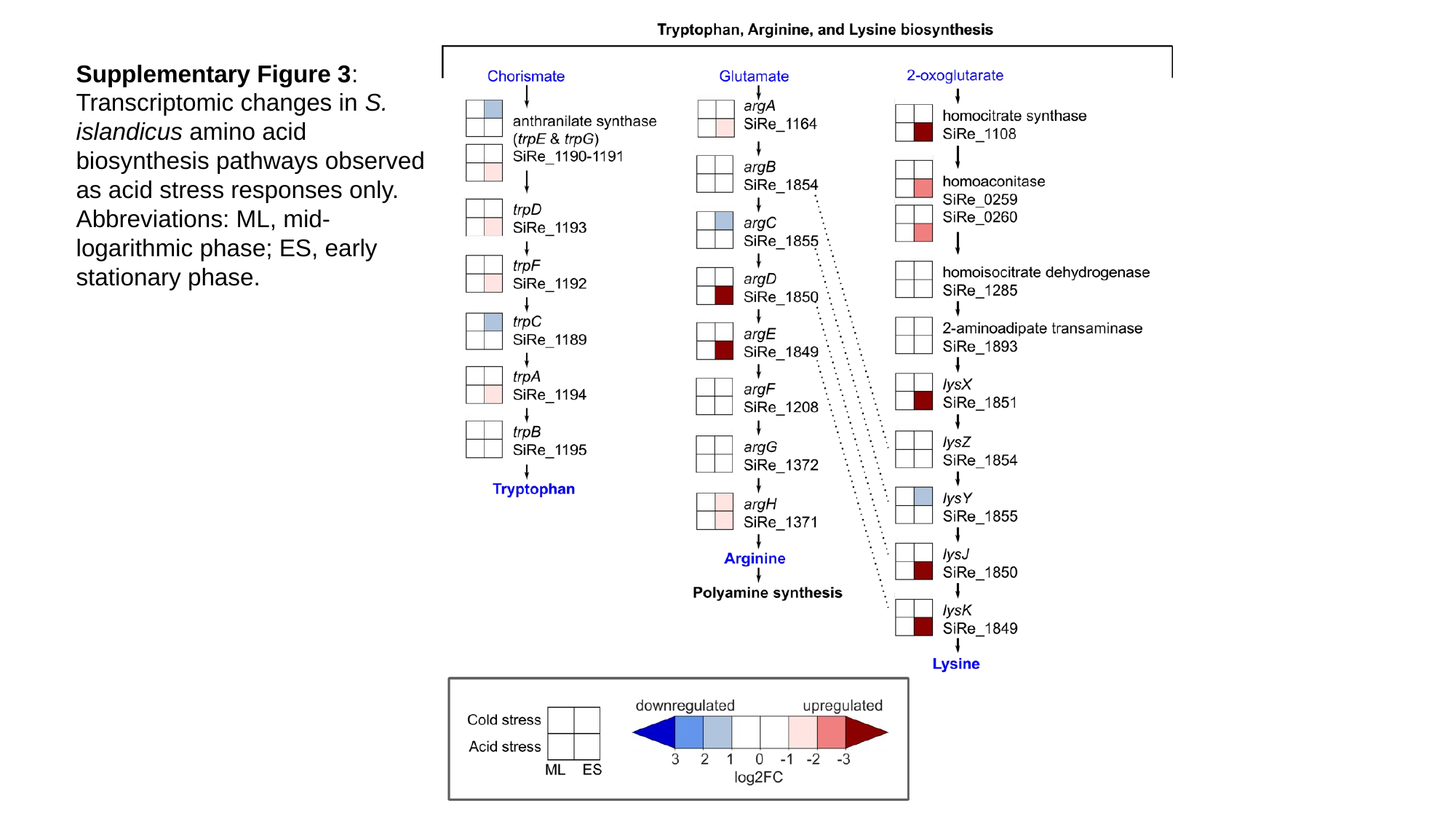

Supplementary Figure 3: Transcriptomic changes in S. islandicus amino acid biosynthesis pathways observed as acid stress responses only. Abbreviations: ML, mid-logarithmic phase; ES, early stationary phase.

## Slide 5
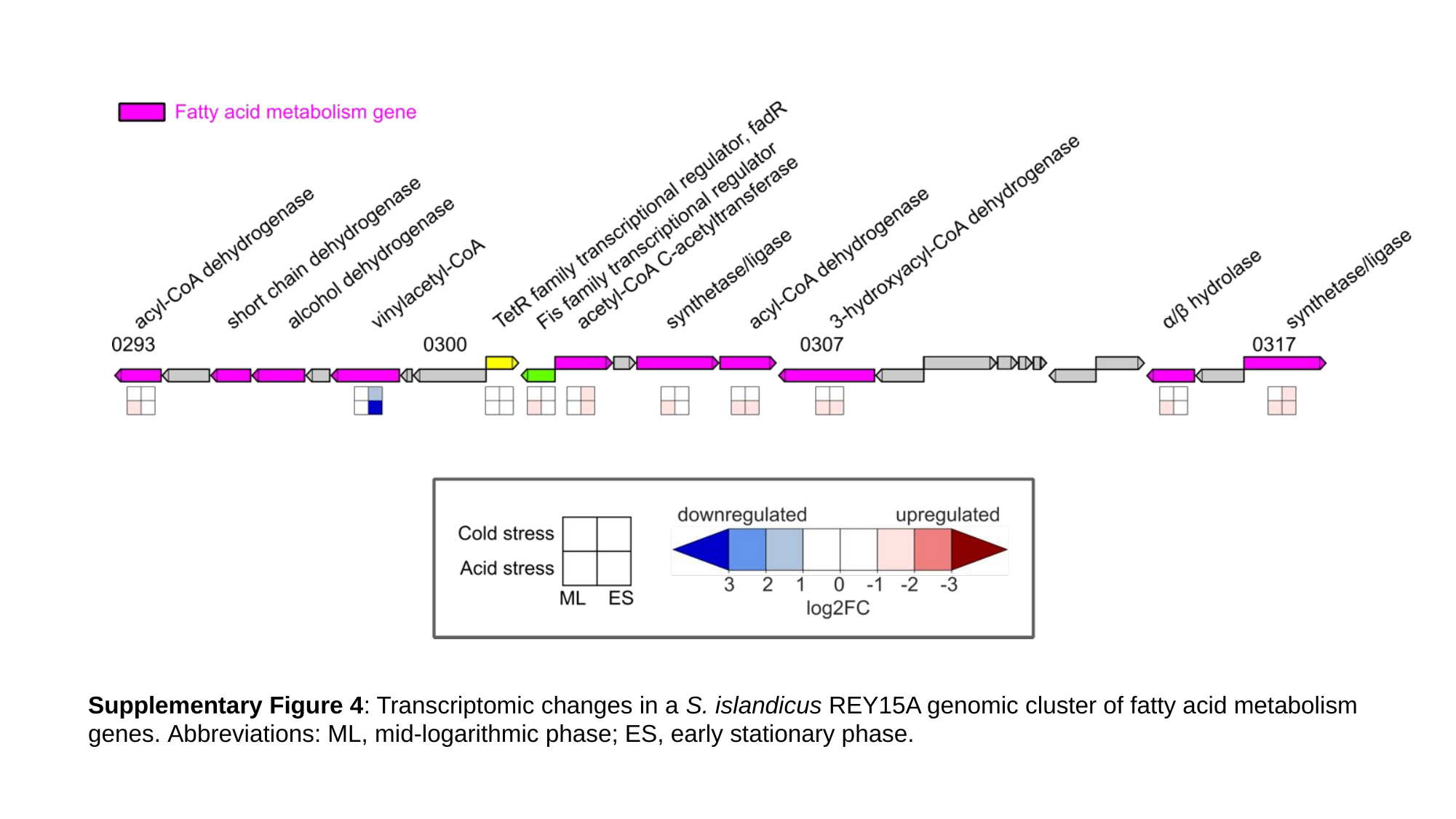

Supplementary Figure 4: Transcriptomic changes in a S. islandicus REY15A genomic cluster of fatty acid metabolism genes. Abbreviations: ML, mid-logarithmic phase; ES, early stationary phase.
